# Voice as a Biomarker of Pediatric Health: A Scoping Review

**DOI:** 10.3390/children11060684

**Published:** 2024-06-04

**Authors:** Hannah Paige Rogers, Anne Hseu, Jung Kim, Elizabeth Silberholz, Stacy Jo, Anna Dorste, Kathy Jenkins

**Affiliations:** 1 Department of Cardiology, Boston Children’s Hospital, Harvard Medical School, 300 Longwood Avenue, Boston, MA 02115, USA; 2 Department of Otolaryngology, Boston Children’s Hospital, 333 Longwood Ave, Boston, MA 02115, USA; 3 Department of Pediatrics, Boston Children’s Hospital, Boston, MA 02115, USA; 4 Boston Children’s Hospital, 300 Longwood Avenue, Boston, MA 02115, USA

**Keywords:** artificial intelligence, machine learning, pediatric health, vocal biomarkers

## Abstract

The human voice has the potential to serve as a valuable biomarker for the early detection, diagnosis, and monitoring of pediatric conditions. This scoping review synthesizes the current knowledge on the application of artificial intelligence (AI) in analyzing pediatric voice as a biomarker for health. The included studies featured voice recordings from pediatric populations aged 0–17 years, utilized feature extraction methods, and analyzed pathological biomarkers using AI models. Data from 62 studies were extracted, encompassing study and participant characteristics, recording sources, feature extraction methods, and AI models. Data from 39 models across 35 studies were evaluated for accuracy, sensitivity, and specificity. The review showed a global representation of pediatric voice studies, with a focus on developmental, respiratory, speech, and language conditions. The most frequently studied conditions were autism spectrum disorder, intellectual disabilities, asphyxia, and asthma. Mel-Frequency Cepstral Coefficients were the most utilized feature extraction method, while Support Vector Machines were the predominant AI model. The analysis of pediatric voice using AI demonstrates promise as a non-invasive, cost-effective biomarker for a broad spectrum of pediatric conditions. Further research is necessary to standardize the feature extraction methods and AI models utilized for the evaluation of pediatric voice as a biomarker for health. Standardization has significant potential to enhance the accuracy and applicability of these tools in clinical settings across a variety of conditions and voice recording types. Further development of this field has enormous potential for the creation of innovative diagnostic tools and interventions for pediatric populations globally.

## 1. Introduction

The human voice is often referred to as a unique print for each individual. It contains biomarkers that have been linked in the adult literature to various diseases ranging from Parkinson’s disease [1] to dementia, mood disorders, and cancers, such as laryngeal and glottal [2,3,4]. The voice contains complex acoustic markers that depend on respiration, phonation, articulation, and prosody coordination. Recent advances in acoustic analysis technology, especially when coupled with machine learning, have shed new insights into the detection of diseases. As a biomarker, the voice is cost-effective, easy, and safe to collect in low-resource settings. Moreover, the human voice contains not only speech, but other acoustic biomarkers such as cry, cough, and other respiratory sounds.

The existing body of systematic literature on vocal biomarkers for disease detection spans multiple disciplines, including acoustic analysis, machine learning, and application to clinical medicine. Key studies on this topic include a review by Fagherazzi et al. (2023) [5]. Fagherazzi et al. discuss the integration of vocal biomarkers into clinical practice and highlight the potential of non-invasive voice analysis for diagnosing conditions such as Parkinson’s disease, depression, and cardiovascular conditions. Sara et al. (2023) [6] discuss the feasibility of remote health monitoring using voice analysis and demonstrate significant advancements in telehealth, leveraging technology, and machine learning for the detection of conditions such as COVID-19 and chronic obstructive pulmonary disease (COPD). Lastly, Idrisoglu et al. (2022) [7] present a systematic review on various machine learning models used in voice analysis, finding that Support Vector Machine (SVM) and Neural Network models show high accuracy in diagnosing voice disorders. Despite key findings presented in the current literature, several gaps remain. Most studies focus on adult populations leaving a gap in research on vocal biomarkers for the diagnosis of pediatric-specific conditions. Additionally, most studies are region-specific, limiting the generalizability of findings across more diverse populations. Lastly, there is a lack of comprehensive reviews that synthesize findings across many condition types. Our scoping review aims to synthesize existing knowledge on the application of AI in the analysis of pediatric voice as a biomarker for health to foster a deeper understanding of its potential use as an investigative or diagnostic tool within the pediatric clinical setting.

## 2. Materials Methods

### 2.1. Registration and Funding

This scoping review was registered with the Open Science Framework (OSF) to enhance transparency and reproducibility. The review was registered on 24 July 2023 under the OSF registration https://doi.org/10.17605/OSF.IO/SC6MG The full registration details, including the review protocol and objectives, can be accessed at https://osf.io/sc6mg (accessed on 23 May 2024). All phases of this study were supported by the National Institutes of Health grant number: 1OT20D032720-01.

### 2.2. Search Strategy

**Figure 1:**
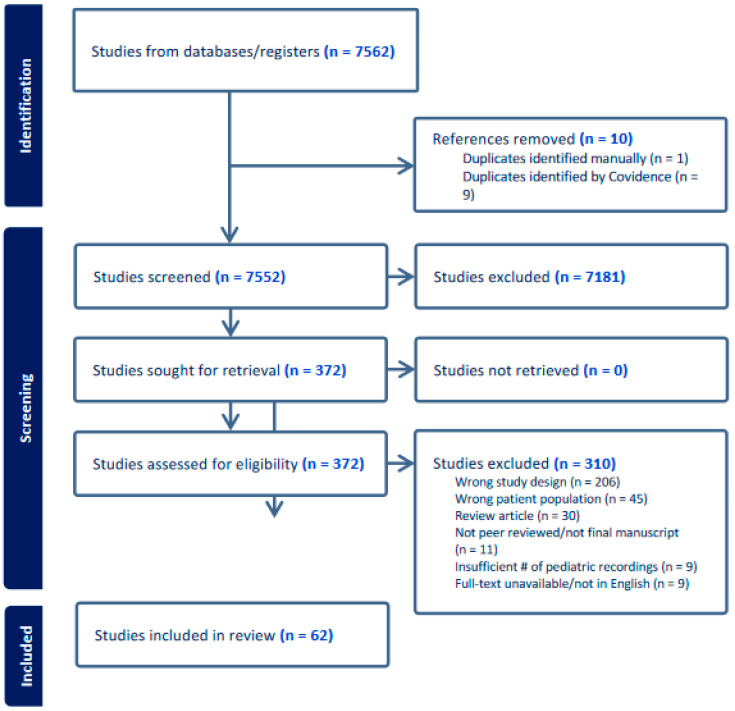
PRISMA flow diagram of study inclusion from study identification, screening, and final inclusion.

Precise searches were conducted to identify relevant keywords and controlled vocabulary for the following concepts: artificial intelligence, voice, pediatrics, and disorders. Controlled vocabulary terms were combined logically by a medical librarian using Boolean logic, with keywords searched in the title and abstract to form a sensitive search strategy. The final search strategy utilized 217 keywords, including 91 related to “artificial intelligence”, 45 related to “voice”, 20 related to “pediatric”, and 61 related to “disorder”, as shown in Appendix A. The original PubMed search was translated into the following databases: Embase, Web of Science Core Collection, and the Cochrane database. Google Scholar and ClinicalTrials.gov were searched in order to pull in the grey literature. All searches were run in May 2023 and de-duplicated in EndNote using the validated deduplication method put forth by Bramer et al. [8]. Results were imported into Covidence, a systematic review software. Titles and abstracts were independently reviewed by two reviewers against pre-defined inclusion criteria. Relevant texts were moved to the full-text review, whereby the same process evaluated PDFs of eligible citations. Conflicting votes were resolved via discussion until the two original reviewers reached a consensus. The PRISMA flow chart of article inclusion is shown in Figure 1.

### 2.3. Inclusion Criteria

Each study was required to include voice recordings in pediatric populations aged 0–17 years. Studies involving both pediatric and adult cohorts were considered on the basis that pediatric data were collected and analyzed separately from adult data. A minimum of 10 pediatric participants were required in each study. All pediatric health conditions were considered except for newborn or infant cry to indicate hunger, discomfort, pain, or sleepiness. Studies were limited to peer-reviewed prospective or retrospective research studies written originally in English and excluded scoping reviews, literature reviews, and meta-analyses. Studies were required to utilize one or more feature extraction methods to produce a vocal dataset and required an analysis of pathological biomarkers contained in voice, cry, or respiratory sounds using one or more machine learning or artificial intelligence models.

### 2.4. Data Extraction

At the final stage, 62 studies met the inclusion criteria (Figure 1). A study was eligible for data extraction after two independent reviewers reached a consensus on its inclusion in the title, abstract, and full-text review phases. Utilizing the data extraction template in Covidence, we customized a tool to collect general study information, study characteristics, participant characteristics, recording sources and data, feature extraction methods, and machine learning or artificial intelligence model types. When available, accuracy, sensitivity, and specificity data were collected for each diagnostic model. These data were synthesized to determine the combination of feature extraction method(s) and artificial intelligence model that results in the highest diagnostic accuracy. Only models that used at least one feature extraction method and presented data for accuracy, sensitivity, and specificity were considered for highest diagnostic accuracy. When more than one model was presented by a study, only the model with the highest diagnostic accuracy was summarized. Four condition groups were determined: developmental conditions, respiratory conditions, speech language conditions, and other non-respiratory conditions. The best models were determined for each condition type within each condition group, with the exception of other non-respiratory conditions.

## 3. Results

### 3.1. Global Representation

**Figure 2:**
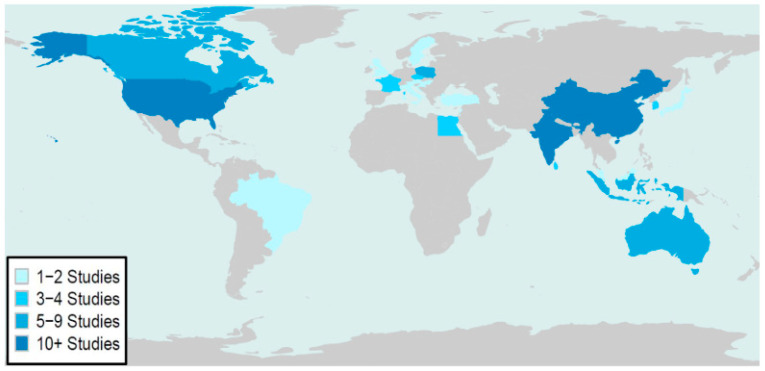
Global heat map of the distribution and frequency of publications included in the scoping review.

Across 62 studies, 25 countries were represented (Appendix B). The global distribution and frequency of publication are shown in Figure 2. Pediatric populations from the United States, India, and China were the most frequently studied. Data primarily represented pediatric populations from North America, Asia, Europe, and Oceania and were less representative of Central and South America, Africa, and the Middle East.

### 3.2. Studies by Year

**Figure 3:**
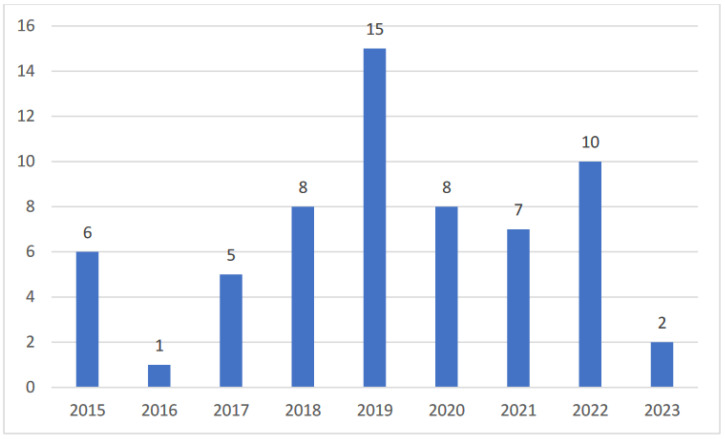
Column graph of publications by year (2015–2023) for all studies included in the scoping review.

This review identified studies published between 2015 and 2023, and data were extracted on 25 May 2023. The number of studies per year is shown in Figure 3, with an average of 7 pediatric voice studies per year between 2015 and 2023 and a peak of 15 publications in 2019.

### 3.3. Funding Sources

Research funding supported 29 studies (46.7%), and 56 different funding sources were represented (Appendix C). Organizations that provided funding to two or more studies included the National Natural Science Foundation of China, Manipal University Jaipur (India), SMART Innovation Centre (USA), Austrian National Bank, National Institute on Deafness and Other Communication Disorders (USA), Austrian Science Fund, Natural Sciences and Engineering Research Council of Canada, and the Bill & Melinda Gates Foundation (USA). Most funding came from public and private organizations from the United States, China, India, and Austria.

### 3.4. Participant Age

**Figure 4:**
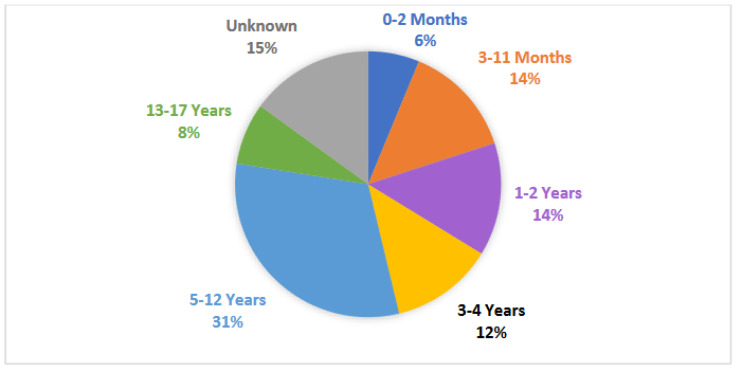
Pie chart of the age distribution of all participants included in the scoping review. Categories: 0–2 months, 3–11 months, 1–2 years, 3–4 years, 5–12 years, 13–17 years, and unknown.

Each study had, on average, 202 participants [range: 12–2268], with a median of 76 participants. A total of 27% of participants (n = 3347) were distinguished by sex, of which 61% were male. School-aged children (ages 5–12 years) were the most commonly studied (25 studies). Newborn (ages 0–2 months), infant (ages 3–11 months), toddler (ages 1–2 years), preschool (ages 3–4 years), school-aged (ages 5–12 years), and teenage (ages 13–17) groups were also represented in at least five studies each as shown in Figure 4. The specific pediatric age group being studied was not defined for 12 studies.

### 3.5. Recording Characteristics

**Figure 5:**
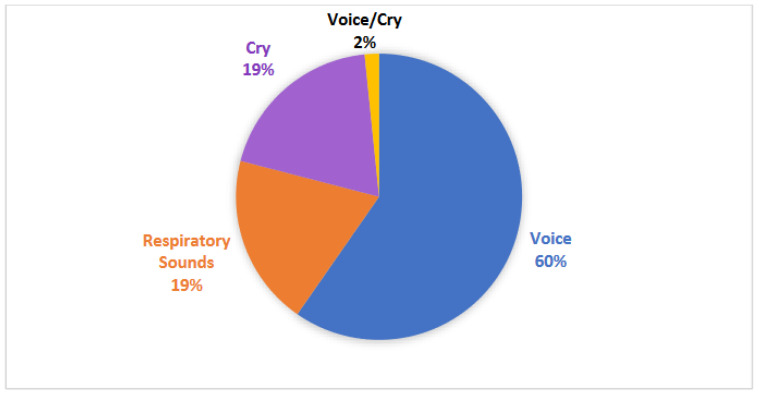
Pie chart of the recording type distribution for all studies included in the scoping review. Categories: voice, respiratory sounds, cry, and voice and cry.

As shown in Figure 5, studies included three types of vocal recordings: voice (38 studies), cry (13 studies), and respiratory sounds (12 studies). The majority of studies (45 studies) collected unique vocal data, while 17 studies utilized 13 different existing datasets to conduct their studies, of which recordings from the Baby Chillanto Infant Cry Database (Mexico) and the LANNA Research Group Child Speech Database (Czech Republic) were the most commonly studied.

### 3.6. Clinical Conditions

Vocal recordings were analyzed, using AI, as a biomarker for 31 clinical conditions, represented in Appendix D. Among these conditions, developmental conditions (21 studies), respiratory conditions (21 studies), speech and language conditions (13 studies), and non-respiratory conditions (7 studies) were represented. The most frequently studied conditions included autism spectrum disorder (ASD) (12 studies), intellectual disabilities (7 studies), asphyxia (7 studies), and asthma (5 studies).

### 3.7. Feature Extraction Methods

Among 62 studies, 33 feature extraction methods were utilized (Appendix E). Mel-Frequency Cepstral Coefficients (MFCCs) were the most utilized feature extraction method (43 studies), followed by Spectral Components (10 studies), Cepstral Coefficients (10 studies), Pitch and Fundamental Frequency (9 studies), and Linear Predictive Coefficients (9 studies).

### 3.8. Artificial Intelligence and Machine Learning Models

Across the studies, 33 artificial intelligence or machine learning models were utilized (Appendix F). The most common AI/ML models were Support Vector Machine (SVM) (34 studies), Neural Network (31 studies), Random Forest (9 studies), Linear Discriminant Analysis (LDA) (7 studies), and K-Nearest Neighbor (KNN) (5 studies).

### 3.9. Model Accuracy

Among 54 studies (excluding those on non-respiratory conditions), 85 models were summarized based on feature extraction methods, AI model types, and their diagnostic accuracy, sensitivity, and specificity. Out of these, 39 models were evaluated and compared for diagnostic accuracy (Appendix H). Fifteen models achieved high diagnostic accuracy: three for developmental conditions (Table 1), eight for respiratory conditions (Table 2), and four for speech–language conditions (Table 3).

For diagnosing developmental conditions, the best models often utilized voice recordings with Mel-Frequency Cepstral Coefficients (MFCCs) and Support Vector Machines (SVM) or Neural Networks. Jayasree (2021) [9] achieved 100% accuracy, sensitivity, and specificity for diagnosing autism spectrum disorder using MFCCs and a Feed-Forward Neural Network.

For respiratory diagnosis, the top models frequently used recordings of coughs, respiratory sounds, and cries. MFCCs were the most common feature extraction method, often combined with the Non-Gaussianity Score. Neural Networks and SVM were the most utilized AI models. Notably, Hariharan (2018) [10] and Gouda (2019) [11] achieved 100% accuracy for diagnosing asphyxia and wheezing, using Improved Binary Dragonfly Optimization and Artificial Neural Networks, respectively.

In the category of speech–language conditions, voice recordings were commonly used, but there was no dominant feature extraction method among the four models with high accuracy. Hariharan (2018) [10] and Barua (2023) [12] developed models with 100% and 99.9% accuracy for detecting deafness and speech–language impairment, using Improved Binary Dragonfly Optimization and SVM.

Overall, MFCCs combined with SVM resulted in the highest diagnostic accuracy across all condition groups and types.

## 4. Discussion

The human voice contains unique, complex acoustic markers that vary depending on one’s coordination between respiration, phonation, articulation, and prosody. As technology progresses, especially in artificial intelligence and acoustic analysis, voice is emerging as a cost-effective, non-invasive, and accessible biomarker for the detection of pathologies. Our primary objective was to determine what is currently known about using pediatric voice paired with AI models for the early detection, diagnosis, and monitoring of pediatric conditions. This review identified 62 studies that met the inclusion criteria, utilizing pediatric voice, cry, or respiratory sounds for the detection of 31 pediatric conditions among four condition groups, representing pediatric populations from 25 countries.

### 4.1. Developmental Conditions

Twenty-one of the included studies trained and evaluated machine learning algorithms using voice data to classify children with developmental disorders. Speech was the predominantly utilized feature, with studies considering various aspects of speech, including vocal, acoustic, phonetic, and language features. Acoustic features [13,14,15] and phonetic features [16] were extracted to train machine learning algorithms in classifying children with intellectual disabilities. A majority of the included studies centered on training machine learning algorithms to classify children with autism spectrum disorder (and Down Syndrome [9]) using acoustic features [9,17,18,19,20,21], vocal features [22,23], voice prosody features [24], pre-linguistic vocal features [25], and speech features [26,27]. In particular, Wu et al. (2019) [21] focused on acoustic features of crying sounds in children of 2 to 3 years of age, while Pokorny et al. (2017) [28] concentrated on pre-linguistic vocal features in 10-month-old babies. Speech features were also utilized in training machine learning algorithms to classify children with developmental language disorders [29], specific language impairments [30,31], and dyslexia [32,33]. Acoustic and phonetic features were commonly extracted using Mel-Frequency Cepstral Coefficients to train Neural Network or Support Vector Machine algorithms.

### 4.2. Respiratory Conditions

Twenty-one of the included studies focused on the unintentional air movement across vocal cords by cry, cough, or breath. Machine learning techniques characterized infant cries in the setting of asphyxia [34,35,36,37,38]. Spontaneous pediatric coughs are rigorously described through AI methodology [39,40,41,42,43] and analyzed to detect specific clinical entities such as croup [42,44,45], pertussis [45], asthma [46], and pneumonia [40]. Asthma, a common childhood illness, has also been studied through AI analysis of pediatric breath sounds [47,48]. Nearly all respiratory studies utilized Mel-Frequency Cepstral Coefficients to extract features from cough, respiratory sound, and cry recordings which were applied to Neural Network and Support Vector Machine algorithms.

### 4.3. Speech and Language Conditions

The detection and evaluation of voice and speech disorders in children is uniquely challenging due to the intricate nature of speech production and the variability inherent in children’s speech patterns. To address these challenges, researchers have explored a variety of computational approaches leveraging machine learning, neural networks, and signal processing techniques aimed toward the early identification of speech delay [49,50]. Several studies highlight promising methodologies to identify stuttering and specific language impairment (SLI) using acoustic and linguistic features [51,52]. Feature extraction techniques and convolutional neural networks can help to detect hypernasality in children with cleft palates [53,54,55]. Voice acoustic parameters have been developed to identify dysphonia and vocal nodules in children [56,57]. Automatic acoustic analysis can also be used to differentiate typically developing children from those who are hard of hearing, language-delayed, and autistic [58]. Other notable research has utilized deep learning models and computer-aided systems to identify SLI and sigmatism, also known as lisping [59,60,61]. Similar to developmental and respiratory condition diagnosis, Mel-Frequency Cepstral Coefficients were the preferred feature extraction method. However, there was variation in the AI model used. It is notable that across the studies included, each model presented for the diagnosis of speech and language conditions achieved diagnostic accuracy ≥ 86 percent, most with accuracy ≥ 90 percent.

### 4.4. Other Non-Respiratory Conditions

Researchers have explored using voice recordings and AI to identify other non-respiratory genetic or medical conditions, usually based on known characteristics affecting cry, voice, or speech that can lead to a clinical suspicion that a diagnosis is present. A Voice Biometric System was developed using recordings of 15 two-syllable words to identify whether a child has cerebral palsy and the severity of the condition, with potential usefulness to evaluate therapeutic benefit [62]. A hierarchical machine learning model using voice recordings of the standardized PATA speech test was able to identify and grade the level of severity of dysarthria associated with ataxia [56]. Early detection of anxiety and depression using a 3 min Speech Task in 3-to-8-year-olds showed reasonable accuracy when recordings were high quality [63], and multimodal text and audio data were able to discriminate adolescents with depression based on recorded interviews [64]. Recordings of cry sounds have also been evaluated using machine learning and have shown reasonable accuracy in detecting life-threatening sepsis in neonates [65,66] and neonatal opioid withdrawal syndrome [67]. Mel-Frequency Cepstral Coefficients, Cepstral Coefficients, and Harmonic-to-Noise Ratio were most often used for feature extraction and applied to Support Vector Machine algorithms for the detection of non-respiratory conditions. The data are best viewed by condition type, due to the variety of conditions included in this group.

### 4.5. Limitations

This review was restricted to studies published in English, which may not capture the full scope of research in non-English-speaking regions. Additionally, the inclusion criteria required a sample size of at least 10 pediatric participants in each study. Studies with fewer than 10 participants may offer valuable insights, but they did not meet inclusion criteria within this review.

Another limitation of this study was the inherent lack of standardization in feature extraction methods and AI model architectures. This variability makes it challenging to compare results across studies. To identify models with the highest diagnostic accuracy, we designed a strict inclusion criteria for comparison. However, it should be noted that there is considerable nuance in the development of models within the current state of voice diagnostics. Standardizing these methods in this developing field could facilitate more accurate comparisons and lead to more robust meta-analyses in the future.

## 5. Conclusions

This scoping review highlights the current and potential applications of AI in analyzing pediatric voice as a biomarker for health. AI models have been used with pediatric voice data for the early detection, diagnosis, and monitoring of 32 pediatric conditions, including autism spectrum disorder (ASD), intellectual disabilities, asphyxia, and asthma. While most applications focus on developmental, respiratory, and speech and language conditions, this review also explores using pediatric voice analysis for detecting non-respiratory conditions such as anxiety and depression, sepsis, and jaundice.

Research thus far demonstrates the enormous potential of using voice recordings to detect and monitor diseases and conditions in children. The data indicate that using Mel-Frequency Cepstral Coefficients (MFCCs) as a feature extraction method, combined with Neural Networks or Support Vector Machines, results in high diagnostic accuracy across various conditions and vocal recording types. However, the lack of standardization in feature extraction methods and AI model architectures presents a challenge in comparing results across studies. Future research should investigate the use of standardized methods to facilitate more accurate comparisons and robust meta-analyses. Additionally, future research should explore the use of AI diagnosis for pediatric conditions not yet covered in this review.

While most studies have recorded voices in clinical settings, there is potential for using voice recordings as biomarkers in non-clinical settings where children are more comfortable, such as at home or school. Further development in this field could lead to innovative diagnostic tools and interventions for pediatric populations globally.

## Figures and Tables

**Table 1:** Developmental Conditions: Models with Highest Diagnostic Accuracy.

Developmental Conditions: Models with Highest Diagnostic Accuracy							
Study	Condition Type	Recording Type	Feature Extraction Methods	Artificial Intelligence Model	Accuracy	Sensitivity	Specificity
Jayasree 2021	Autism Spectrum Disorder	Voice	Mel Frequency Cepstral Coefficients	Neural Network	100	100	100
Sharma 2020	Intellectual Disability	Voice	Local Binary Patterns	Support Vector Machine	98.7	99	99.2
Ribeiro 2020	Dyslexia	Voice	Local Binary Patterns	Support Vector Machine	94.4	100	80

**Table 2:** Respiratory Conditions: Models with Highest Diagnostic Accuracy.

Respiratory Conditions: Models with Highest Diagnostic Accuracy							
Study	Condition Type	Recording Type	Feature Extraction Methods	Artificial Intelligence Model	Accuracy	Sensitivity	Specificity
Hariharan 2018	Asphyxia	Cry	Linear Predictive Coefficients Nonlinear Entropies Wavelet Packet Transform	Improved Binary Dragonfly Optimization	100	100	100
Gouda 2019	Wheezing	Respiratory	Discrete Wavelet Transform Mel Frequency Cepstral Coefficients Short Time Fourier Transform;	Neural Network	100	100	100
Amrulloh 2015a	Cough Segments	Cough	Mel Frequency Cepstral Coefficients Non-Gaussianity Score Shannon Entropy Zero Crossing Rate	Linear Discriminant Analysis	97.4	97.5	92.8
Amrulloh 2018	Wet/Dry Cough	Cough	Formant Frequency Mel Frequency Cepstral Coefficients Non-Gaussianity Score Shannon Entropy	Neural Network	96.4	96.5	96.6
Khalilzad 2022a	Respiratory Distress Syndrome	Cry	Cepstral Coefficients Harmonic-to-Noise Ratio	Support Vector Machine	95.3	95	95
Amrulloh 2015b	Asthma	Cough	Formant Frequency Mel Frequency Cepstral Coefficients Non-Gaussianity Score Shannon Entropy	Neural Network	94.4	100	88.9
Sharan 2017	Croup	Cough	Mel Frequency Cepstral Coefficients Cochleagram Image Feature	Support Vector Machine	91.2	88.4	91.6
Sharan 2021	Whooping Cough	Respiratory	Mel Frequency Cepstral Coefficients Cochleagram Image Feature	Neural Network	90.5	95.2	85.7

**Table 3:** Speech Language Conditions: Models with Highest Diagnostic Accuracy.

Speech Language Conditions: Models with Highest Diagnostic Accuracy							
Study	Condition Type	Recording Type	Feature Extraction Methods	Artificial Intelligence Model	Accuracy	Sensitivity	Specificity
Hariharan 2018	Deafness	Cry	Linear Predictive Coefficients Non-Linear Entropy Wavelet Packet Transform	Improved Binary Dragonfly Optimization	100	100	100
Barua 2023	Speech Language Impairment	Voice	Wavelet Packet Decomposition	Support Vector Machine	99.9	99.9	99.9
Miodonska 2016	Sigmatism	Voice	Delta Coefficients Mel Frequency Cepstral Coefficients	Support Vector Machine	94.5	96	93
Suthar 2022	Speech Disorder	Voice	Landmark Analysis	Linear Discriminant Analysis	93	94	92

## Data Availability

The raw data supporting the conclusions of this article will be made available by the authors on request.

## Appendix A

A table showing the Boolean search strategy employed to compile studies from PubMed, Cochrane Database, Embase, Web of Science, ClinicalTrials.Gov, and Google Scholar.

**Table A1:** Boolean search strategy.

Searches run: 18 May 2023	
Publication date filter: 1 January 2015–18 May 2023	
Language filter: None	
Search	Results
PubMed	3467
#1: (“Artificial Intelligence”[mesh] OR “artificial intelligence”[tiab] OR “machine learning”[tiab] OR “deep learning”[tiab] OR “computational intelligence”[tiab] OR “computer reasoning”[tiab] OR “computer vision system”[tiab] OR “computer vision systems”[tiab] OR “transfer learning”[tiab] OR “hierarchical learning”[tiab] OR “learning from labeled data”[tiab] OR “support vector network”[tiab] OR “support vector networks”[tiab] OR “support vector machine”[tiab] OR “support vector machines”[tiab] OR “ambient intelligence”[tiab] OR “automated reasoning”[tiab] OR “computer heuristics”[tiab] OR “cognitive technology”[tiab] OR “cognitive technologies”[tiab] OR “cognitive computing”[tiab] OR “cognitive robotics”[tiab] OR “optical character recognition”[tiab] OR “robotic process automation”[tiab] OR “machine intelligence”[tiab] OR “artificial superintelligence”[tiab] OR “artificial general intelligence”[tiab] OR “machine reasoning”[tiab] OR “automated inference”[tiab] OR “heuristic algorithm”[tiab] OR “heuristic algorithms”[tiab] OR metaheuristic*[tiab] OR meta-heuristic*[tiab] OR “data mining”[tiab] OR “neural network”[tiab] OR “neural networks”[tiab] OR “neural networking”[tiab] OR “feature learning”[tiab] OR “feature extraction”[tiab] OR “Bayesian learning”[tiab] OR “Bayesian inference”[tiab] OR “multicriteria decision analysis”[tiab] OR “unsupervised learning”[tiab] OR “semi-supervised learning”[tiab] OR “semi supervised learning”[tiab] OR “ANN analysis”[tiab] OR “ANN analyses”[tiab] OR “ANN method”[tiab] OR “ANN methods”[tiab] OR “ANN model”[tiab] OR “ANN models”[tiab] OR “ANN modeling”[tiab] OR “ANN methodology”[tiab] OR “ANN methodologies”[tiab] OR “artificial NN”[tiab] OR “ANN technique”[tiab] OR “ANN techniques”[tiab] OR “ANN output”[tiab] OR “ANN outputs”[tiab] OR “ANN approach”[tiab] OR “network learning”[tiab] OR “random forest”[tiab] OR “relevance vector machine”[tiab] OR “relevance vector machines”[tiab] OR “online analytical processing”[tiab] OR “sentiment analysis”[tiab] OR “sentiment analyses”[tiab] OR “opinion mining”[tiab] OR “sentiment classification”[tiab] OR “sentiment classifications”[tiab] OR “fuzzy logic”[tiab] OR “natural language processing”[tiab] OR “expert system”[tiab] OR “expert systems”[tiab] OR “biological ontology”[tiab] OR “biological ontologies”[tiab] OR “biomedical ontology”[tiab] OR “biomedical ontologies”[tiab] OR “biologic ontology”[tiab] OR “biologic ontologies”[tiab] OR “computer simulation”[tiab] OR “computer simulations”[tiab] OR “Multidimensional Voice Program”[tiab] OR MDVP[tiab] OR “k-nearest neighbor”[tiab] OR “supervised learning algorithm”[tiab] OR “swarm intelligent”[tiab] OR “Swarm intelligence”[tiab] OR “firefly algorithm”[tiab] OR bootstrap*[tiab] OR “fuzzy data fusion”[tiab])	374,243
#2: (Voice[mesh] OR “Voice Recognition”[mesh] OR Speech[mesh] OR Acoustics[mesh] OR Phonation[mesh] OR Linguistics[mesh] OR “Vocal Cords”[mesh] OR Singing[mesh] OR Crying[mesh] OR voice*[tiab] OR speech*[tiab] OR acoustic*[tiab] OR phonat*[tiab] OR vox[tiab] OR language*[tiab] OR linguistic*[tiab] OR speak*[tiab] OR sing[tiab] OR singing[tiab] OR vocal*[tiab] OR respirat*[tiab] OR articulat*[tiab] OR prosody[tiab] OR pitch[tiab] OR “fundamental frequency”[tiab] OR “fundamental frequencies”[tiab] OR f0[tiab] OR “disturbance index”[tiab] OR jitter*[tiab] OR shimmer*[tiab] OR “vocal intensity”[tiab] OR “acoustic voice quality index”[tiab] OR AVQI[tiab] OR “speech-to-noise ratio”[tiab] OR “Speech to noise ratio”[tiab] OR “speech to noise ratios”[tiab] OR “speech-to-noise ratios”[tiab] OR “sound pressure level”[tiab] OR “sound pressure levels”[tiab] OR “cepstral peak prominence”[tiab] OR resonance*[tiab] OR dysphonia[tiab] OR laryngeal[tiab] OR larynx[tiab] OR laryn[tiab] OR banking[tiab] OR communicat*[tiab] OR cry[tiab] OR crying[tiab] OR cries[tiab] OR squeal*[tiab] OR babble[tiab] OR babbling[tiab])	2,220,825
#3: (Child[mesh] OR Infant[mesh] OR Adolescent[mesh] OR Pediatrics[mesh] OR “Child Health”[mesh] OR “Infant Health”[mesh] OR “Adolescent Health”[mesh] OR Minors[mesh] OR “Young Adult”[mesh] OR child*[tiab] OR pediatric*[tiab] OR paediatric*[tiab] OR infant[tiab] OR infants[tiab] OR neonat*[tiab] OR newborn*[tiab] OR baby[tiab] OR babies[tiab] OR toddler*[tiab] OR adolescen*[tiab] OR teen*[tiab] OR youth*[tiab] OR juvenile*[tiab] OR “emerging adult”[tiab] OR “emerging adults”[tiab] OR “young adult”[tiab] OR “young adults”[tiab] OR minor[tiab] OR minors[tiab]) 5,428,993	5,428,993
#4: (“Neurodevelopmental Disorders”[mesh] OR “Communication Disorders”[mesh] OR “Speech-Language Pathology”[mesh] OR “Voice Disorders”[mesh] OR “Speech Disorders”[mesh] OR “Speech Production Measurement”[mesh] OR “Laryngeal Diseases”[mesh] OR “Genetic Diseases, Inborn”[mesh] OR “Cleft Lip”[mesh] OR “Cleft Palate”[mesh] OR “Hearing Aids”[mesh:noexp] OR “Cochlear Implants”[mesh] OR “Cochlear Implantation”[mesh] OR “Sleep Apnea, Obstructive”[mesh] OR “Hearing Loss”[mesh] OR “Language Development Disorders”[mesh] OR Asthma[mesh] OR “Rhinitis, Allergic, Seasonal”[mesh] OR “Diabetes Mellitus, Type 1”[mesh] OR “Whooping Cough”[mesh] OR Dyslexia[mesh] OR disorder*[tiab] OR patholog*[tiab] OR disease*[tiab] OR malform*[tiab] OR abnormal*[tiab] OR language[tiab] OR autism[tiab] OR autistic[tiab] OR ASD[tiab] OR syndrome*[tiab] OR syndromic[tiab] OR “Developmental language disorder”[tiab] OR “vocal cord dysfunction”[tiab] OR “dysfunctional vocal cord”[tiab] OR “dysfunctional vocal cords”[tiab] OR “vocal fold lesion”[tiab] OR “vocal fold lesions”[tiab] OR “cleft lip”[tiab] OR “cleft lips”[tiab] OR “cleft palate”[tiab] OR “cleft palates”[tiab] OR “laryngotracheal reconstruction”[tiab] OR “reconstructed larynx”[tiab] OR “reconstructed trachea”[tiab] OR “laryngotracheal reconstructions”[tiab] OR “hearing impairment”[tiab] OR “hearing impairments”[tiab] OR “hearing loss”[tiab] OR deaf[tiab] OR deafness[tiab] OR “hearing impaired”[tiab] OR “cochlear implant”[tiab] OR “cochlear implants”[tiab] OR “cochlear implantation”[tiab] OR “cochlear implantations”[tiab] OR “obstructive sleep apnea”[tiab] OR “obstructive sleep apneas”[tiab] OR OSA[tiab] OR asthma*[tiab] OR “seasonal allergy”[tiab] OR “seasonal allergies”[tiab] OR “allergic rhinitis”[tiab] OR “allergic rhinosinusitis”[tiab] OR “hay fever”[tiab] OR “Type 1 diabetes”[tiab] OR “type 1 diabetic”[tiab] OR “type 1 diabetics”[tiab] OR “juvenile onset diabetes”[tiab] OR “insulin dependent diabetes”[tiab] OR pertussis[tiab] OR “whooping cough”[tiab] OR dyslexia[tiab] OR dyslexic[tiab] OR biomark*[tiab] OR healthy[tiab] OR prevent*[tiab] OR screen*[tiab] OR develop*[tiab] OR detect*[tiab] OR early[tiab] OR diagnos*[tiab])	16,466,029
#5: #1 AND #2 AND #3 AND #4	4828
#6: #5, 2015–Present	3467
Cochrane Database	318
#1 MeSH descriptor: [Artificial Intelligence] explode all trees	2832
#2 (“artificial intelligence” OR “machine learning” OR “deep learning” OR “computational intelligence” OR “computer reasoning” OR “computer vision system” OR “computer vision systems” OR “transfer learning” OR “hierarchical learning” OR “learning from labeled data” OR “support vector network” OR “support vector networks” OR “support vector machine” OR “support vector machines” OR “ambient intelligence” OR “automated reasoning” OR “computer heuristics” OR “cognitive technology” OR “cognitive technologies” OR “cognitive computing” OR “cognitive robotics” OR “optical character recognition” OR “robotic process automation” OR “machine intelligence” OR “artificial superintelligence” OR “artificial general intelligence” OR “machine reasoning” OR “automated inference” OR “heuristic algorithm” OR “heuristic algorithms” OR metaheuristic* OR meta-heuristic* OR “data mining” OR “neural network” OR “neural networks” OR “neural networking” OR “feature learning” OR “feature extraction” OR “Bayesian learning” OR “Bayesian inference” OR “multicriteria decision analysis” OR “unsupervised learning” OR “semi-supervised learning” OR “semi supervised learning” OR “ANN analysis” OR “ANN analyses” OR “ANN method” OR “ANN methods” OR “ANN model” OR “ANN models” OR “ANN modeling” OR “ANN methodology” OR “ANN methodologies” OR “artificial NN” OR “ANN technique” OR “ANN techniques” OR “ANN output” OR “ANN outputs” OR “ANN approach” OR “network learning” OR “random forest” OR “relevance vector machine” OR “relevance vector machines” OR “online analytical processing” OR “sentiment analysis” OR “sentiment analyses” OR “opinion mining” OR “sentiment classification” OR “sentiment classifications” OR “fuzzy logic” OR “natural language processing” OR “expert system” OR “expert systems” OR “biological ontology” OR “biological ontologies” OR “biomedical ontology” OR “biomedical ontologies” OR “biologic ontology” OR “biologic ontologies” OR “computer simulation” OR “computer simulations” OR “Multidimensional Voice Program” OR MDVP OR “k-nearest neighbor” OR “supervised learning algorithm” OR “swarm intelligent” OR “Swarm intelligence” OR “firefly algorithm” OR bootstrap* OR “fuzzy data fusion”):ti,ab,kw	11,352
#3 #1 OR #2	12,450
#4 MeSH descriptor: [Voice] explode all trees	572
#5 MeSH descriptor: [Voice Recognition] explode all trees	0
#6 MeSH descriptor: [Speech] explode all trees	1325
#7 MeSH descriptor: [Acoustics] explode all trees	530
#8 MeSH descriptor: [Phonation] explode all trees	246
#9 MeSH descriptor: [Linguistics] explode all trees	1779
#10 MeSH descriptor: [Vocal Cords] explode all trees	171
#11 MeSH descriptor: [Singing] explode all trees	93
#12 MeSH descriptor: [Crying] explode all trees	418
#13 (voice* OR speech* OR acoustic* OR phonat* OR vox OR language* OR linguistic* OR speak* OR sing OR singing OR vocal* OR respirat* OR articulat* OR prosody OR pitch OR “fundamental frequency” OR “fundamental frequencies” OR f0 OR “disturbance index” OR jitter* OR shimmer* OR “vocal intensity” OR “acoustic voice quality index” OR AVQI OR “speech-to-noise ratio” OR “Speech to noise ratio” OR “speech to noise ratios” OR “speech-to-noise ratios” OR “sound pressure level” OR “sound pressure levels” OR “cepstral peak prominence” OR resonance* OR dysphonia OR laryngeal OR larynx OR laryn OR banking OR communicat* OR cry OR crying OR cries OR squeal* OR babble OR babbling):ti,ab,kw	203,049
#14 #4 OR #5 OR #6 OR #7 OR #8 OR #9 OR #10 OR #11 OR #12 OR #13	204,107
#15 MeSH descriptor: [Child] explode all trees	77,718
#16 MeSH descriptor: [Infant] explode all trees	41,571
#17 MeSH descriptor: [Adolescent] explode all trees	125,309
#18 MeSH descriptor: [Pediatrics] explode all trees	1178
#19 MeSH descriptor: [Child Health] explode all trees	307
#20 MeSH descriptor: [Infant Health] explode all trees	84
#21 MeSH descriptor: [Adolescent Health] explode all trees	84
#22 MeSH descriptor: [Minors] explode all trees	11
#23 MeSH descriptor: [Young Adult] explode all trees	84,591
#24 (child* OR pediatric* OR paediatric* OR infant OR infants OR neonat* OR newborn* OR baby OR babies OR toddler* OR adolescen* OR teen* OR youth* OR juvenile* OR “emerging adult” OR “emerging adults” OR “young adult” OR “young adults” OR minor OR minors):ti,ab,kw	415,796
#25 #15 OR #16 OR #17 OR #18 OR #19 OR #20 OR #21 OR #22 OR #23 OR #24	415,809
#26 MeSH descriptor: [Neurodevelopmental Disorders] explode all trees	9895
#27 MeSH descriptor: [Communication Disorders] explode all trees	2347
#28 MeSH descriptor: [Speech-Language Pathology] explode all trees	108
#29 MeSH descriptor: [Voice Disorders] explode all trees	703
#30 MeSH descriptor: [Speech Disorders] explode all trees	1214
#31 MeSH descriptor: [Speech Production Measurement] explode all trees	215
#32 MeSH descriptor: [Laryngeal Diseases] explode all trees	1629
#33 MeSH descriptor: [Genetic Diseases, Inborn] explode all trees	16,887
#34 MeSH descriptor: [Cleft Lip] explode all trees	365
#35 MeSH descriptor: [Cleft Palate] explode all trees	445
#36 MeSH descriptor: [Hearing Aids] explode all trees	592
#37 MeSH descriptor: [Cochlear Implants] explode all trees	216
#38 MeSH descriptor: [Cochlear Implantation] explode all trees	151
#39 MeSH descriptor: [Sleep Apnea, Obstructive] explode all trees	2627
#40 MeSH descriptor: [Hearing Loss] explode all trees	1609
#41 MeSH descriptor: [Language Development Disorders] explode all trees	252
#42 MeSH descriptor: [Asthma] explode all trees	14,957
#43 MeSH descriptor: [Rhinitis, Allergic, Seasonal] explode all trees	2174
#44 MeSH descriptor: [Diabetes Mellitus, Type 1] explode all trees	6747
#45 MeSH descriptor: [Whooping Cough] explode all trees	406
#46 MeSH descriptor: [Dyslexia] explode all trees	366
#47 (disorder* OR patholog* OR disease* OR malform* OR abnormal* OR language OR autism OR autistic OR ASD OR syndrome* OR syndromic OR “Developmental language disorder” OR “vocal cord dysfunction” OR “dysfunctional vocal cord” OR “dysfunctional vocal cords” OR “vocal fold lesion” OR “vocal fold lesions” OR “cleft lip” OR “cleft lips” OR “cleft palate” OR “cleft palates” OR “laryngotracheal reconstruction” OR “reconstructed larynx” OR “reconstructed trachea” OR “laryngotracheal reconstructions” OR “hearing impairment” OR “hearing impairments” OR “hearing loss” OR deaf OR deafness OR “hearing impaired” OR “cochlear implant” OR “cochlear implants” OR “cochlear implantation” OR “cochlear implantations” OR “obstructive sleep apnea” OR “obstructive sleep apneas” OR OSA OR asthma* OR “seasonal allergy” OR “seasonal allergies” OR “allergic rhinitis” OR “allergic rhinosinusitis” OR “hay fever” OR “Type 1 diabetes” OR “type 1 diabetic” OR “type 1 diabetics” OR “juvenile onset diabetes” OR “insulin dependent diabetes” OR pertussis OR “whooping cough” OR dyslexia OR dyslexic OR biomark* OR healthy OR prevent* OR screen* OR develop* OR detect* OR early OR diagnos*):ti,ab,kw	1,297,671
#48 #26 OR #27 OR #28 OR #29 OR #30 OR #31 OR #32 OR #33 OR #34 OR #35 OR #36 OR #37 OR #38 OR #39 OR #40 OR #41 OR #42 OR #43 OR #44 OR #45 OR #46 OR #47	1,303,057
#49 #3 AND #14 AND #25 AND #48	417
From 1 January 2015–18 May 2023: 4 Reviews, 314 Trials	
Embase	4262
#1: ‘artificial intelligence’/exp OR ‘machine learning’/exp OR ‘natural language processing’/exp OR ‘artificial intelligence’:ab,ti,kw OR ‘machine learning’:ab,ti,kw OR ‘deep learning’:ab,ti,kw OR ‘computational intelligence’:ab,ti,kw OR ‘computer reasoning’:ab,ti,kw OR ‘computer vision system’:ab,ti,kw OR ‘computer vision systems’:ab,ti,kw OR ‘transfer learning’:ab,ti,kw OR ‘hierarchical learning’:ab,ti,kw OR ‘learning from labeled data’:ab,ti,kw OR ‘support vector network’:ab,ti,kw OR ‘support vector networks’:ab,ti,kw OR ‘support vector machine’:ab,ti,kw OR ‘support vector machines’:ab,ti,kw OR ‘ambient intelligence’:ab,ti,kw OR ‘automated reasoning’:ab,ti,kw OR ‘computer heuristics’:ab,ti,kw OR ‘cognitive technology’:ab,ti,kw OR ‘cognitive technologies’:ab,ti,kw OR ‘cognitive computing’:ab,ti,kw OR ‘cognitive robotics’:ab,ti,kw OR ‘optical character recognition’:ab,ti,kw OR ‘robotic process automation’:ab,ti,kw OR ‘machine intelligence’:ab,ti,kw OR ‘artificial superintelligence’:ab,ti,kw OR ‘artificial general intelligence’:ab,ti,kw OR ‘machine reasoning’:ab,ti,kw OR ‘automated inference’:ab,ti,kw OR ‘heuristic algorithm’:ab,ti,kw OR ‘heuristic algorithms’:ab,ti,kw OR metaheuristic*:ab,ti,kw OR ‘meta heuristic*’:ab,ti,kw OR ‘data mining’:ab,ti,kw OR ‘neural network’:ab,ti,kw OR ‘neural networks’:ab,ti,kw OR ‘neural networking’:ab,ti,kw OR ‘feature learning’:ab,ti,kw OR ‘feature extraction’:ab,ti,kw OR ‘bayesian learning’:ab,ti,kw OR ‘bayesian inference’:ab,ti,kw OR ‘multicriteria decision analysis’:ab,ti,kw OR ‘unsupervised learning’:ab,ti,kw OR ‘semi-supervised learning’:ab,ti,kw OR ‘semi supervised learning’:ab,ti,kw OR ‘ann analysis’:ab,ti,kw OR ‘ann analyses’:ab,ti,kw OR ‘ann method’:ab,ti,kw OR ‘ann methods’:ab,ti,kw OR ‘ann model’:ab,ti,kw OR ‘ann models’:ab,ti,kw OR ‘ann modeling’:ab,ti,kw OR ‘ann methodology’:ab,ti,kw OR ‘ann methodologies’:ab,ti,kw OR ‘artificial nn’:ab,ti,kw OR ‘ann technique’:ab,ti,kw OR ‘ann techniques’:ab,ti,kw OR ‘ann output’:ab,ti,kw OR ‘ann outputs’:ab,ti,kw OR ‘ann approach’:ab,ti,kw OR ‘network learning’:ab,ti,kw OR ‘random forest’:ab,ti,kw OR ‘relevance vector machine’:ab,ti,kw OR ‘relevance vector machines’:ab,ti,kw OR ‘online analytical processing’:ab,ti,kw OR ‘sentiment analysis’:ab,ti,kw OR ‘sentiment analyses’:ab,ti,kw OR ‘opinion mining’:ab,ti,kw OR ‘sentiment classification’:ab,ti,kw OR ‘sentiment classifications’:ab,ti,kw OR ‘fuzzy logic’:ab,ti,kw OR ‘natural language processing’:ab,ti,kw OR ‘expert system’:ab,ti,kw OR ‘expert systems’:ab,ti,kw OR ‘biological ontology’:ab,ti,kw OR ‘biological ontologies’:ab,ti,kw OR ‘biomedical ontology’:ab,ti,kw OR ‘biomedical ontologies’:ab,ti,kw OR ‘biologic ontology’:ab,ti,kw OR ‘biologic ontologies’:ab,ti,kw OR ‘computer simulation’:ab,ti,kw OR ‘computer simulations’:ab,ti,kw OR ‘multidimensional voice program’:ab,ti,kw OR mdvp:ab,ti,kw OR ‘k-nearest neighbor’:ab,ti,kw OR ‘supervised learning algorithm’:ab,ti,kw OR ‘swarm intelligent’:ab,ti,kw OR ‘swarm intelligence’:ab,ti,kw OR ‘firefly algorithm’:ab,ti,kw OR bootstrap*:ab,ti,kw OR ‘fuzzy data fusion’:ab,ti,kw	559, 152
#2: ‘speech and language’/exp OR ‘voice recognition’/exp OR ‘speech’/exp OR ‘acoustics’/exp OR ‘singing’/exp OR ‘crying’/exp OR ‘vocal cord’/exp OR voice*:ab,ti,kw OR speech*:ab,ti,kw OR acoustic*:ab,ti,kw OR phonat*:ab,ti,kw OR vox:ab,ti,kw OR language*:ab,ti,kw OR linguistic*:ab,ti,kw OR speak*:ab,ti,kw OR sing:ab,ti,kw OR singing:ab,ti,kw OR vocal*:ab,ti,kw OR respirat*:ab,ti,kw OR articulat*:ab,ti,kw OR prosody:ab,ti,kw OR pitch:ab,ti,kw OR ‘fundamental frequency’:ab,ti,kw OR ‘fundamental frequencies’:ab,ti,kw OR f0:ab,ti,kw OR ‘disturbance index’:ab,ti,kw OR jitter*:ab,ti,kw OR shimmer*:ab,ti,kw OR ‘vocal intensity’:ab,ti,kw OR ‘acoustic voice quality index’:ab,ti,kw OR avqi:ab,ti,kw OR ‘speech-to-noise ratio’:ab,ti,kw OR ‘speech to noise ratio’:ab,ti,kw OR ‘speech to noise ratios’:ab,ti,kw OR ‘speech-to-noise ratios’:ab,ti,kw OR ‘sound pressure level’:ab,ti,kw OR ‘sound pressure levels’:ab,ti,kw OR ‘cepstral peak prominence’:ab,ti,kw OR resonance*:ab,ti,kw OR dysphonia:ab,ti,kw OR laryngeal:ab,ti,kw OR larynx:ab,ti,kw OR laryn:ab,ti,kw OR banking:ab,ti,kw OR communicat*:ab,ti,kw OR cry:ab,ti,kw OR crying:ab,ti,kw OR cries:ab,ti,kw OR squeal*:ab,ti,kw OR babble:ab,ti,kw OR babbling:ab,ti,kw	2,852,403
#3: ‘pediatrics’/exp OR ‘child’/exp OR ‘adolescent’/exp OR ‘juvenile’/de OR ‘adolescent health’/exp OR ‘child health’/exp OR child*:ab,ti,kw OR pediatric*:ab,ti,kw OR paediatric*:ab,ti,kw OR infant:ab,ti,kw OR infants:ab,ti,kw OR neonat*:ab,ti,kw OR newborn*:ab,ti,kw OR baby:ab,ti,kw OR babies:ab,ti,kw OR toddler*:ab,ti,kw OR adolescen*:ab,ti,kw OR teen*:ab,ti,kw OR youth*:ab,ti,kw OR juvenile*:ab,ti,kw OR ‘emerging adult’:ab,ti,kw OR ‘emerging adults’:ab,ti,kw OR ‘young adult’:ab,ti,kw OR ‘young adults’:ab,ti,kw OR minor:ab,ti,kw OR minors:ab,ti,kw	5,784,184
#4: ‘speech disorder’/exp OR ‘mental disease’/exp OR ‘communication disorder’/exp OR ‘voice disorder’/exp OR ‘voice disorder assessment’/exp OR ‘speech analysis’/exp OR ‘larynx disorder’/exp OR ‘congenital disorder’/exp OR ‘cleft lip with or without cleft palate’/exp OR ‘palate malformation’/exp OR ‘hearing aid’/de OR ‘cochlea prosthesis’/exp OR ‘sleep disordered breathing’/exp OR ‘hearing impairment’/exp OR ‘developmental language disorder’/exp OR ‘asthma’/exp OR ‘allergic rhinitis’/exp OR ‘insulin dependent diabetes mellitus’/de OR ‘pertussis’/de OR ‘dyslexia’/exp OR disorder*:ab,ti,kw OR patholog*:ab,ti,kw OR disease*:ab,ti,kw OR malform*:ab,ti,kw OR abnormal*:ab,ti,kw OR language:ab,ti,kw OR autism:ab,ti,kw OR autistic:ab,ti,kw OR asd:ab,ti,kw OR syndrome*:ab,ti,kw OR syndromic:ab,ti,kw OR ‘developmental language disorder’:ab,ti,kw OR ‘vocal cord dysfunction’:ab,ti,kw OR ‘dysfunctional vocal cord’:ab,ti,kw OR ‘dysfunctional vocal cords’:ab,ti,kw OR ‘vocal fold lesion’:ab,ti,kw OR ‘vocal fold lesions’:ab,ti,kw OR ‘cleft lip’:ab,ti,kw OR ‘cleft lips’:ab,ti,kw OR ‘cleft palate’:ab,ti,kw OR ‘cleft palates’:ab,ti,kw OR ‘laryngotracheal reconstruction’:ab,ti,kw OR ‘reconstructed larynx’:ab,ti,kw OR ‘reconstructed trachea’:ab,ti,kw OR ‘laryngotracheal reconstructions’:ab,ti,kw OR ‘hearing impairment’:ab,ti,kw OR ‘hearing impairments’:ab,ti,kw OR ‘hearing loss’:ab,ti,kw OR deaf:ab,ti,kw OR deafness:ab,ti,kw OR ‘hearing impaired’:ab,ti,kw OR ‘cochlear implant’:ab,ti,kw OR ‘cochlear implants’:ab,ti,kw OR ‘cochlear implantation’:ab,ti,kw OR ‘cochlear implantations’:ab,ti,kw OR ‘obstructive sleep apnea’:ab,ti,kw OR ‘obstructive sleep apneas’:ab,ti,kw OR osa:ab,ti,kw OR asthma*:ab,ti,kw OR ‘seasonal allergy’:ab,ti,kw OR ‘seasonal allergies’:ab,ti,kw OR ‘allergic rhinitis’:ab,ti,kw OR ‘allergic rhinosinusitis’:ab,ti,kw OR ‘hay fever’:ab,ti,kw OR ‘type 1 diabetes’:ab,ti,kw OR ‘type 1 diabetic’:ab,ti,kw OR ‘type 1 diabetics’:ab,ti,kw OR ‘juvenile onset diabetes’:ab,ti,kw OR ‘insulin dependent diabetes’:ab,ti,kw OR pertussis:ab,ti,kw OR ‘whooping cough’:ab,ti,kw OR dyslexia:ab,ti,kw OR dyslexic:ab,ti,kw OR biomark*:ab,ti,kw OR healthy:ab,ti,kw OR prevent*:ab,ti,kw OR screen*:ab,ti,kw OR develop*:ab,ti,kw OR detect*:ab,ti,kw OR early:ab,ti,kw OR diagnos*:ab,ti,kw	22,970,561
#5: #1 AND #2 AND #3 AND #4	5542
#6: #5 AND [2015–2023]/py	4262
Web of Science Core Collection	2992
#1: TI = (“artificial intelligence” OR “machine learning” OR “deep learning” OR “computational intelligence” OR “computer reasoning” OR “computer vision system” OR “computer vision systems” OR “transfer learning” OR “hierarchical learning” OR “learning from labeled data” OR “support vector network” OR “support vector networks” OR “support vector machine” OR “support vector machines” OR “ambient intelligence” OR “automated reasoning” OR “computer heuristics” OR “cognitive technology” OR “cognitive technologies” OR “cognitive computing” OR “cognitive robotics” OR “optical character recognition” OR “robotic process automation” OR “machine intelligence” OR “artificial superintelligence” OR “artificial general intelligence” OR “machine reasoning” OR “automated inference” OR “heuristic algorithm” OR “heuristic algorithms” OR metaheuristic* OR meta-heuristic* OR “data mining” OR “neural network” OR “neural networks” OR “neural networking” OR “feature learning” OR “feature extraction” OR “Bayesian learning” OR “Bayesian inference” OR “multicriteria decision analysis” OR “unsupervised learning” OR “semi-supervised learning” OR “semi supervised learning” OR “ANN analysis” OR “ANN analyses” OR “ANN method” OR “ANN methods” OR “ANN model” OR “ANN models” OR “ANN modeling” OR “ANN methodology” OR “ANN methodologies” OR “artificial NN” OR “ANN technique” OR “ANN techniques” OR “ANN output” OR “ANN outputs” OR “ANN approach” OR “network learning” OR “random forest” OR “relevance vector machine” OR “relevance vector machines” OR “online analytical processing” OR “sentiment analysis” OR “sentiment analyses” OR “opinion mining” OR “sentiment classification” OR “sentiment classifications” OR “fuzzy logic” OR “natural language processing” OR “expert system” OR “expert systems” OR “biological ontology” OR “biological ontologies” OR “biomedical ontology” OR “biomedical ontologies” OR “biologic ontology” OR “biologic ontologies” OR “computer simulation” OR “computer simulations” OR “Multidimensional Voice Program” OR MDVP OR “k-nearest neighbor” OR “supervised learning algorithm” OR “swarm intelligent” OR “Swarm intelligence” OR “firefly algorithm” OR bootstrap* OR “fuzzy data fusion”)	559,410
#2: AB = (“artificial intelligence” OR “machine learning” OR “deep learning” OR “computational intelligence” OR “computer reasoning” OR “computer vision system” OR “computer vision systems” OR “transfer learning” OR “hierarchical learning” OR “learning from labeled data” OR “support vector network” OR “support vector networks” OR “support vector machine” OR “support vector machines” OR “ambient intelligence” OR “automated reasoning” OR “computer heuristics” OR “cognitive technology” OR “cognitive technologies” OR “cognitive computing” OR “cognitive robotics” OR “optical character recognition” OR “robotic process automation” OR “machine intelligence” OR “artificial superintelligence” OR “artificial general intelligence” OR “machine reasoning” OR “automated inference” OR “heuristic algorithm” OR “heuristic algorithms” OR metaheuristic* OR meta-heuristic* OR “data mining” OR “neural network” OR “neural networks” OR “neural networking” OR “feature learning” OR “feature extraction” OR “Bayesian learning” OR “Bayesian inference” OR “multicriteria decision analysis” OR “unsupervised learning” OR “semi-supervised learning” OR “semi supervised learning” OR “ANN analysis” OR “ANN analyses” OR “ANN method” OR “ANN methods” OR “ANN model” OR “ANN models” OR “ANN modeling” OR “ANN methodology” OR “ANN methodologies” OR “artificial NN” OR “ANN technique” OR “ANN techniques” OR “ANN output” OR “ANN outputs” OR “ANN approach” OR “network learning” OR “random forest” OR “relevance vector machine” OR “relevance vector machines” OR “online analytical processing” OR “sentiment analysis” OR “sentiment analyses” OR “opinion mining” OR “sentiment classification” OR “sentiment classifications” OR “fuzzy logic” OR “natural language processing” OR “expert system” OR “expert systems” OR “biological ontology” OR “biological ontologies” OR “biomedical ontology” OR “biomedical ontologies” OR “biologic ontology” OR “biologic ontologies” OR “computer simulation” OR “computer simulations” OR “Multidimensional Voice Program” OR MDVP OR “k-nearest neighbor” OR “supervised learning algorithm” OR “swarm intelligent” OR “Swarm intelligence” OR “firefly algorithm” OR bootstrap* OR “fuzzy data fusion”)	1,263,010
#3: #1 OR #2	1,377,387
#4: TI = (voice* OR speech* OR acoustic* OR phonat* OR vox OR language* OR linguistic* OR speak* OR sing OR singing OR vocal* OR respirat* OR articulat* OR prosody OR pitch OR “fundamental frequency” OR “fundamental frequencies” OR f0 OR “disturbance index” OR jitter* OR shimmer* OR “vocal intensity” OR “acoustic voice quality index” OR AVQI OR “speech-to-noise ratio” OR “Speech to noise ratio” OR “speech to noise ratios” OR “speech-to-noise ratios” OR “sound pressure level” OR “sound pressure levels” OR “cepstral peak prominence” OR resonance* OR dysphonia OR laryngeal OR larynx OR laryn OR banking OR communicat* OR cry OR crying OR cries OR squeal* OR babble OR babbling)	1,654,988
#5: AB = (voice* OR speech* OR acoustic* OR phonat* OR vox OR language* OR linguistic* OR speak* OR sing OR singing OR vocal* OR respirat* OR articulat* OR prosody OR pitch OR “fundamental frequency” OR “fundamental frequencies” OR f0 OR “disturbance index” OR jitter* OR shimmer* OR “vocal intensity” OR “acoustic voice quality index” OR AVQI OR “speech-to-noise ratio” OR “Speech to noise ratio” OR “speech to noise ratios” OR “speech-to-noise ratios” OR “sound pressure level” OR “sound pressure levels” OR “cepstral peak prominence” OR resonance* OR dysphonia OR laryngeal OR larynx OR laryn OR banking OR communicat* OR cry OR crying OR cries OR squeal* OR babble OR babbling)	3,869,279
#6: #4 OR #5	4,678,033
#7: TI = (child* OR pediatric* OR paediatric* OR infant OR infants OR neonat* OR newborn* OR baby OR babies OR toddler* OR adolescen* OR teen* OR youth* OR juvenile* OR “emerging adult” OR “emerging adults” OR “young adult” OR “young adults” OR minor OR minors)	2,197,895
#8: AB = (child* OR pediatric* OR paediatric* OR infant OR infants OR neonat* OR newborn* OR baby OR babies OR toddler* OR adolescen* OR teen* OR youth* OR juvenile* OR “emerging adult” OR “emerging adults” OR “young adult” OR “young adults” OR minor OR minors)	2,469,292
#9: #7 OR #8	3,568,507
#10: TI = (disorder* OR patholog* OR disease* OR malform* OR abnormal* OR language OR autism OR autistic OR ASD OR syndrome* OR syndromic OR “Developmental language disorder” OR “vocal cord dysfunction” OR “dysfunctional vocal cord” OR “dysfunctional vocal cords” OR “vocal fold lesion” OR “vocal fold lesions” OR “cleft lip” OR “cleft lips” OR “cleft palate” OR “cleft palates” OR “laryngotracheal reconstruction” OR “reconstructed larynx” OR “reconstructed trachea” OR “laryngotracheal reconstructions” OR “hearing impairment” OR “hearing impairments” OR “hearing loss” OR deaf OR deafness OR “hearing impaired” OR “cochlear implant” OR “cochlear implants” OR “cochlear implantation” OR “cochlear implantations” OR “obstructive sleep apnea” OR “obstructive sleep apneas” OR OSA OR asthma* OR “seasonal allergy” OR “seasonal allergies” OR “allergic rhinitis” OR “allergic rhinosinusitis” OR “hay fever” OR “Type 1 diabetes” OR “type 1 diabetic” OR “type 1 diabetics” OR “juvenile onset diabetes” OR “insulin dependent diabetes” OR pertussis OR “whooping cough” OR dyslexia OR dyslexic OR biomark* OR healthy OR prevent* OR screen* OR develop* OR detect* OR early OR diagnos*)	8,242,145
#11: AB = (disorder* OR patholog* OR disease* OR malform* OR abnormal* OR language OR autism OR autistic OR ASD OR syndrome* OR syndromic OR “Developmental language disorder” OR “vocal cord dysfunction” OR “dysfunctional vocal cord” OR “dysfunctional vocal cords” OR “vocal fold lesion” OR “vocal fold lesions” OR “cleft lip” OR “cleft lips” OR “cleft palate” OR “cleft palates” OR “laryngotracheal reconstruction” OR “reconstructed larynx” OR “reconstructed trachea” OR “laryngotracheal reconstructions” OR “hearing impairment” OR “hearing impairments” OR “hearing loss” OR deaf OR deafness OR “hearing impaired” OR “cochlear implant” OR “cochlear implants” OR “cochlear implantation” OR “cochlear implantations” OR “obstructive sleep apnea” OR “obstructive sleep apneas” OR OSA OR asthma* OR “seasonal allergy” OR “seasonal allergies” OR “allergic rhinitis” OR “allergic rhinosinusitis” OR “hay fever” OR “Type 1 diabetes” OR “type 1 diabetic” OR “type 1 diabetics” OR “juvenile onset diabetes” OR “insulin dependent diabetes” OR pertussis OR “whooping cough” OR dyslexia OR dyslexic OR biomark* OR healthy OR prevent* OR screen* OR develop* OR detect* OR early OR diagnos*)	20,002,177
#12: #10 OR #11	23,974,526
#13: #3 AND #6 AND #9 AND #12	3933
#14: #3 AND #6 AND #9 AND #12 and 2023 or 2022 or 2021 or 2020 or 2019 or 2018 or 2017 or 2016 or 2015 (Publication Years)	2992
ClinicalTrials.Gov	12
#1: “artificial intelligence” OR “machine learning” | speech OR Voice OR acoustic | Child	12
Google Scholar	600
#1: (“artificial intelligence” OR “machine learning” OR “deep learning”) AND (voice OR speech OR language) AND (child OR children OR pediatrics) AND (Disorder OR disorders OR disease OR diseases)	100
#2: (“artificial intelligence” OR “machine learning” OR “deep learning”) AND (respiration OR pitch OR vocal OR speaking) AND (child OR children OR pediatrics) AND (Disorder OR disorders OR disease OR diseases)	100
#3: (“artificial intelligence” OR “machine learning” OR “deep learning”) AND (dysphonia OR larynx OR communication OR cry) AND (child OR children OR pediatrics) AND (Disorder OR disorders OR disease OR diseases)	100
#4: (“artificial intelligence” OR “machine learning” OR “deep learning”) AND (voice OR speech OR language OR acoustic) AND (child OR children OR pediatrics) AND (autism OR autistic OR syndrome OR syndromic)	100
#5: (“artificial intelligence” OR “machine learning” OR “deep learning”) AND (voice OR speech OR language OR acoustic) AND (child OR children OR pediatrics) AND (dyslexia OR deaf OR “hearing impairment” OR “vocal cord”)	100
#6: (“artificial intelligence” OR “machine learning” OR “deep learning”) AND (voice OR speech OR language OR acoustic) AND (child OR children OR pediatrics) AND (screen OR detecting OR prevention OR diagnosis)	100
Total for deduplication:	11,651
* Indicates Boolean wildcard search	

## Appendix B

A table listing the country associated with each study and their reference number.

**Table A2:** Study by Country.

Country	Study	Reference #
Australia [4]	Porter 2019	[41]
	Sharan 2017	[42]
	Sharan 2019	[44]
	Sharan 2021	[45]
Austria [1]	Pokorny 2022	[25]
Brazil [1]	Ribeiro 2020	[33]
Canada [4]	Khalilzad 2022	[65]
	Khalilzad 2022	[66]
	Salehian Matikolaie 2021	[68]
	Sharma 2020	[30]
China [6]	Chen 2023	[16]
	Wang 2019a	[54]
	Wang 2019b	[55]
	Wu 2019	[21]
	Zhang 2020	[29]
	Zhang 2022	[64]
Croatia [1]	Mazic 2015	[48]
Czech Republic [1]	Barua 2023	[12]
	Kotarba 2020	[59]
Egypt [1]	Badreldine 2018	[34]
	Gouda 2019	[11]
France [2]	Bokov 2015	[47]
	Deng 2017	[18]
Hungary [1]	Tulics 2018	[57]
India [7]	Aggarwal 2020a	[13]
	Aggarwal 2018	[14]
	Aggarwal 2020	[15]
	Dubey 2018	[53]
	Jayasree 2021	[9]
	Moharir 2017	[62]
	Sharma 2022	[31]
Indonesia [4]	Amrulloh 2015	[39]
	Amrulloh 2015	[43]
	Amrulloh 2018	[40]
	Nafisah 2019	[69]
Italy [1]	Tartarisco 2021	[56]
Japan [1]	Nakai 2017	[24]
Lebanon [1]	Salehian Matikolaie 2020	[68]
Malaysia [1]	Hariharan 2018	[10]
Palestine [1]	Khalilzad 2022	[66]
Poland [4]	Kotarba 2020	[59]
	Miodonska 2016	[60]
	Szklanny 2019	[70]
	Woloshuk 2018	[61]
Singapore [2]	Balamurali 2021	[52]
	Hee 2019	[46]
South Korea [2]	Lee 2020	[19]
	Lee 2022	[20]
Sri Lanka [2]	Kariyawasam 2019	[32]
	Wijesinghe 2019	[27]
Sweden [1]	Pokorny 2017	[28]
Turkey [1]	Satar 2022	[38]
United Kingdom [1]	Alharbi 2018	[51]
USA [12]	Asgari 2021	[22]
	Chi 2022	[26]
	Cho 2019	[17]
	Ji 2021	[35]
	Ji 2019	[36]
	MacFarlane 2022	[23]
	Manigault 2022	[67]
	McGinnis 2019	[63]
	Onu 2019	[37]
	Sadeghian 2015	[49]
	Suthar 2022	[50]
	VanDam 2015	[58]

## Appendix C

A table listing the funding source, funding country, study, and reference number for each study that stated a funding source within their respective publication.

**Table A3:** Funding source by study and country.

Country	Funding Source	Study	Reference #
Australia	ResApp Health	Porter 2019	[41]
Austria	Austrian National Bank (Oesterreichische Nationalbank)	Pokorny 2017	[28]
		Pokorny 2022	[25]
	Austrian Science Fund	Pokorny 2017	[28]
		Pokorny 2022	[25]
Brazil	FAPEMIG	Ribeiro 2020	[33]
	Universidade Federal de Ouro Preto	Ribeiro 2020	[33]
Canada	Natural Sciences and Engineering Research Council of Canada	Khalilzad 2022	[65]
		Salehian Matikolaie 2020	[68]
		Khalilzad 2022	[66]
	SMART Technologies	Balamurali 2021	[52]
		Hee 2019	[46]
China	Anhui Provincial Natural Science Research Project of Colleges and Universities	Wu 2019	[21]
	Dulwich College Suzhou	Aggarwal 2020	[15]
	Guangzhou City Scientific Research Project	Zhang 2020	[29]
	National Key R&D Program of China	Chen 2023	[16]
		Zhang 2022	[64]
	National Natural Science Foundation of China	Chen 2023	[16]
		Wang 2019	[55]
		Wu 2019	[21]
	Natural Science Foundation of Anhui Province	Wu 2019	[21]
	Science and Technology Plan Transfer Payment Project of Sichuan Province	Zhang 2022	[64]
	Sichuan University	Zhang 2022	[64]
	Sun Yat-sen University	Zhang 2020	[29]
	Yibin Municipal People Government University	Zhang 2022	[64]
Egypt	Alexandria University	Badreldine 2018	[34]
European Union	EU H2020 Program	Pokorny 2017	[28]
India	Government of India (Department of Biotechnology)	Dubey 2018	[53]
	Government of India (Ministry of Human Resource Development)	Dubey 2018	[53]
	Manipal University	Aggarwal 2018	[14]
		Aggarwal 2020	[15]
	NorthCap University	Aggarwal 2018	[14]
Japan	Japan Society for the Promotion of Science	Nakai 2017	[24]
Poland	Polish Ministry of Science	Woloshuk 2018	[61]
	Silesian University of Technology	Wu 2019	[21]
Saudi Arabia	King Saud University	Alharbi 2018	[51]
	Saudi Ministry of Education	Alharbi 2018	[51]
	The IsDB Transform Fund	Chi 2022	[26]
South Korea	Institute of Information and Communications Technology Planning and Evaluation	Lee 2022	[20]
Sri Lanka	Sri Lanka Institute of Information Technology	Wijesinghe 2019	[27]
Sweden	Ban of Sweden Tercentenary Foundation	Pokorny 2017	[28]
	Swedish Research Council	Pokorny 2017	[28]
United States	Auburn University	Suthar 2022	[50]
	Lucile Packard Foundation	Chi 2022	[26]
	Bill & Melinda Gates Foundation	Amrulloh 2015	[39]
		Chi 2022	[26]
		Khalilzad 2022	[65]
		Salehian Matikolaie 2020	[68]
	BioTechMed-Graz	Pokorny 2017	[28]
	Bio-X Center	Chi 2022	[26]
	Brown University	Manigault 2022	[67]
	Coulter Foundation	Chi 2022	[26]
	Hartwell Foundation	Chi 2022	[26]
	Lucile Packard Foundation	Chi 2022	[26]
	National Institute on Deafness and Other Communication Disorders	VanDam 2015	[58]
	National Institutes of Health	Asgari 2021	[22]
		Chi 2022	[26]
	National Science Foundation	Chi 2022	[26]
	Old Dominion University—Virginia Modeling	Aggarwal 2020	[15]
	Plough Foundation	VanDam 2015	[58]
	Stanford University	Chi 2022	[26]
	Weston Havens Foundation	Chi 2022	[26]

## Appendix D

A table listing the condition group and condition type being analyzed by each study included in the scoping review and their associated reference number.

**Table A4:** Condition type and group by study.

Condition Group	Condition Type	Study	Reference #
Developmental Condition	Autism Spectrum Disorder	Asgari 2021	[22]
		Chi 2022	[26]
		Cho 2019	[17]
		Deng 2017	[18]
		Jayasree 2021	[9]
		Lee 2020	[19]
		Lee 2022	[20]
		MacFarlane 2022	[23]
		Nakai 2017	[24]
		Pokorny 2017	[28]
		Wijesinghe 2019	[27]
		Wu 2019	[21]
	Dyslexia	Kariyawasam 2019	[32]
		Ribeiro 2020	[33]
	Intellectual Disability	Aggarwal 2020	[13]
		Aggarwal 2018	[14]
		Aggarwal 2020	[15]
		Chen 2023	[16]
		Sharma 2020	[30]
		Sharma 2022	[31]
		Zhang 2020	[29]
Non-Respiratory Condition	Anxiety/Depression	McGinnis 2019	[63]
		Zhang 2022	[64]
	Ataxia	Tartarisco 2021	[56]
	Cerebral Palsy	Nafisah 2019	[69]
	Down Syndrome	Jayasree 2021	[9]
	Fragile X Syndrome	Pokorny 2022	[25]
	Jaundice	Hariharan 2018	[10]
	Neonatal Opioid Withdrawal Syndrome (NOWS)	Manigault 2022	[67]
	Rett Syndrome	Pokorny 2022	[25]
	Sepsis	Khalilzad 2022	[65]
		Khalilzad 2022	[66]
Respiratory Conditions	Asphyxia	Badreldine 2018	[34]
		Hariharan 2018	[10]
		Ji 2021	[35]
		Ji 2019	[36]
		Moharir 2017	[62]
		Onu 2019	[37]
		Satar 2022	[38]
	Asthma	Amrulloh 2015	[43]
		Balamurali 2021	[52]
		Hee 2019	[46]
		Mazic 2015	[48]
		Porter 2019	[41]
	Croup	Sharan 2017	[42]
		Sharan 2019	[44]
	Lower Respiratory Tract Infection	Balamurali 2021	[52]
	Pneumonia	Amrulloh 2015	[43]
		Porter 2019	[41]
	Respiratory Distress Syndrome	Khalilzad 2022	[66]
		Salehian Matikolaie 2020	[68]
	Upper Respiratory Tract Infection	Balamurali 2021	[52]
	Wet/Dry Cough	Amrulloh 2018	[40]
	Wheezing	Bokov 2015	[47]
		Gouda 2019	[11]
		Mazic 2015	[48]
	Whooping Cough (Pertussis)	Sharan 2021	[45]
Speech–Language Pathology	Deafness	Hariharan 2018	[10]
		Ji 2021	[35]
	Dysphonia	Tulics 2018	[57]
	Hearing Loss	VanDam 2015	[58]
	Hypernasality	Dubey 2018	[53]
		Wang 2019	[54]
		Wang 2019	[55]
	Pediatric Speech Delay	Sadeghian 2015	[49]
	Sigmatism	Miodonska 2016	[60]
		Woloshuk 2018	[61]
	Speech Disorder	Suthar 2022	[50]
	Speech Language Impairment	Barua 2023	[12]
		Kotarba 2020	[59]
	Stuttering	Alharbi 2018	[51]
	Vocal Nodules	Szklanny 2019	[70]

## Appendix E

A table listing the feature extraction method utilized by each respective study and their associated reference number.

**Table A5:** Feature extraction method by study.

Feature Extraction Method	Study	Reference #
AlexNet	Zhang 2022	[64]
Cepstral Coefficients	Aggarwal 2020	[15]
	Asgari 2021	[22]
	Deng 2017	[18]
	Hee 2019	[46]
	Khalilzad 2022	[65]
	Khalilzad 2022	[66]
	MacFarlane 2022	[23]
	Manigault 2022	[67]
	Salehian Matikolaie 2020	[68]
	Wang 2019	[54]
Cochleagram Image Feature (CIF)	Sharan 2017	[42]
	Sharan 2019	[44]
	Sharan 2021	[45]
Delta Coefficients	MacFarlane 2022	[23]
	Miodonska 2016	[60]
	Nakai 2017	[24]
Discrete Cosine Series Coefficients (DCSC)	Sadeghian 2015	[49]
Discrete Cosine Transformation Coefficients (DCTC)	Sadeghian 2015	[49]
Discrete Wavelet Mel-Cepstral Coefficient	Wu 2019	[21]
Discrete Wavelet Transform	Badreldine 2018	[34]
	Gouda 2019	[11]
eGeMAPS	Lee 2020	[19]
Energy	Amrulloh 2018	[40]
	Pokorny 2022	[25]
	Pokorny 2017	[28]
	Salehian Matikolaie 2020	[68]
	Satar 2022	[38]
Entropy	Amrulloh 2015	[39]
	Amrulloh 2015	[43]
	Satar 2022	[38]
	Tulics 2018	[57]
Fast Fourier Tranaformation (FTT)	Nafisah 2019	[69]
	Wijesinghe 2019	[27]
Formant Frequency	Amrulloh 2015	[43]
	Amrulloh 2018	[40]
	Cho 2019	[17]
	Wang 2019	[54]
Glottal-to-Noise Excitation Ratio (GNE)	Jayasree 2021	[9]
Harmonic-to-Noise Ratio (HNR)	Asgari 2021	[22]
	Jayasree 2021	[9]
	Khalilzad 2022	[66]
	MacFarlane 2022	[23]
	Pokorny 2017	[28]
	Tartarisco 2021	[56]
Landmark (LM) Analysis	Suthar 2022	[50]
	Tulics 2018	[57]
Linear Predictive Coefficients (LPC)	Aggarwal 2020	[13]
	Aggarwal 2018	[14]
	Aggarwal 2020	[15]
	Amrulloh 2018	[40]
	Chen 2023	[16]
	Hariharan 2018	[10]
	Onu 2019	[37]
	Wang 2019	[54]
	Wu 2019	[21]
Local Binary Patterns (LBP)	Sharma 2020	[30]
Mel-Frequency Cepstral Coefficients (MFCCs)	Aggarwal 2020	[13]
	Aggarwal 2018	[14]
	Aggarwal 2020	[15]
	Alharbi 2018	[51]
	Amrulloh 2015	[39]
	Amrulloh 2015	[43]
	Amrulloh 2018	[40]
	Badreldine 2018	[34]
	Balamurali 2021	[52]
	Chen 2023	[16]
	Chi 2022	[26]
	Cho 2019	[17]
	Dubey 2018	[53]
	Gouda 2019	[11]
	Hee 2019	[46]
	Jayasree 2021	[9]
	Ji 2019	[36]
	Kariyawasam 2019	[32]
	Khalilzad 2022	[65]
	Kotarba 2020	[59]
	Lee 2022	[20]
	Mazic 2015	[48]
	McGinnis 2019	[63]
	Miodonska 2016	[60]
	Moharir 2017	[62]
	Nafisah 2019	[69]
	Onu 2019	[37]
	Pokorny 2022	[25]
	Porter 2019	[41]
	Ribeiro 2020	[33]
	Sadeghian 2015	[49]
	Salehian Matikolaie 2020	[68]
	Sharan 2017	[42]
	Sharan 2019	[44]
	Sharan 2021	[45]
	Tartarisco 2021	[56]
	Tulics 2018	[57]
	Wang 2019	[54]
	Wijesinghe 2019	[27]
	Woloshuk 2018	[61]
	Wu 2019	[21]
Non-Linear Entropies	Hariharan 2018	[10]
Non-Gaussianity Score	Amrulloh 2015	[39]
	Amrulloh 2015	[43]
	Amrulloh 2018	[40]
Pitch and Fundamental Frequency (F0)	Amrulloh 2018	[40]
	Cho 2019	[17]
	Ji 2021	[35]
	MacFarlane 2022	[23]
	McGinnis 2019	[63]
	Nakai 2017	[24]
	Pokorny 2022	[25]
	Tartarisco 2021	[56]
	Tulics 2018	[57]
Short-Time Fourier Transform (STFT)	Gouda 2019	[11]
Signal-to-Noise Ratio (SNR)	Jayasree 2021	[9]
Spectral Components	Asgari 2021	[22]
	Chi 2022	[26]
	McGinnis 2019	[63]
	Nafisah 2019	[69]
	Pokorny 2022	[25]
	Ribeiro 2020	[33]
	Satar 2022	[38]
	Tartarisco 2021	[56]
	Wang 2019	[55]
	Woloshuk 2018	[61]
Statistical Measures	Amrulloh 2018	[40]
	Kotarba 2020	[59]
	Pokorny 2017	[28]
	Woloshuk 2018	[61]
Wavelet Packet Decomposition	Barua 2023	[12]
Wavelet Packet Transform-Based Energy	Hariharan 2018	[10]
Wavelet Transform	Wu 2019	[21]
Weighted Linear Predictive Cepstral Coefficients	Aggarwal 2020	[13]
Zero-Crossing Rate (ZCR)	Amrulloh 2015	[39]
	Amrulloh 2015	[43]
	Amrulloh 2018	[40]
	Chi 2022	[26]
	Cho 2019	[17]
	McGinnis 2019	[63]
	Nafisah 2019	[69]
	Satar 2022	[38]

## Appendix F

A table listing the artificial intelligence or machine learning model utilized by each respective study and their associated reference number.

**Table A6:** Artificial intelligence model by study.

Artificial Intelligence Model	Study	Reference #
AdaBoost	Chen 2023	[16]
Automated Language Measures	MacFarlane 2022	[23]
Back-Propagation Neural Network	Wang 2019	[55]
Bidirectional Long Short-Term Memory	Balamurali 2021	[52]
	Lee 2020	[19]
	Lee 2022	[20]
Extreme Gradient Boosting (XGBoost)	Suthar 2022	[50]
	Zhang 2020	[29]
Extreme Learning Machine	Hariharan 2018	[10]
Gaussian Mixture Model	Hee 2019	[46]
Generative Adversarial Networks	Deng 2017	[18]
Hidden Markov Models	Sadeghian 2015	[49]
Improved Binary Dragonfly Optimization Algorithm	Hariharan 2018	[10]
K-Means Algorithm	Satar 2022	[38]
K-Nearest Neighbor	Aggarwal 2020	[13]
	Gouda 2019	[11]
	Kariyawasam 2019	[32]
	Khalilzad 2022	[65]
	Tartarisco 2021	[56]
Linear Discriminant Analysis	Aggarwal 2020	[13]
	Amrulloh 2015	[39]
	Cho 2019	[17]
	Sharan 2019	[44]
	Suthar 2022	[50]
	VanDam 2015	[58]
	Woloshuk 2018	[61]
Linear Regression Model	Amrulloh 2018	[40]
	McGinnis 2019	[63]
	Sharan 2017	[42]
Multilayer Perceptron	Khalilzad 2022	[66]
Naïve Bayes	Chen 2023	[16]
	Gouda 2019	[11]
	Tartarisco 2021	[56]
Neural Network (Feedforward, Recurrent, Long Short-Term Memory, Convolutional)	Aggarwal 2018	[14]
	Aggarwal 2020	[13]
	Amrulloh 2015	[15]
	Amrulloh 2015	[43]
	Amrulloh 2018	[40]
	Balamurali 2021	[52]
	Chi 2022	[26]
	Gouda 2019	[11]
	Jayasree 2021	[9]
	Ji 2019	[36]
	Ji 2021	[35]
	Kariyawasam 2019	[32]
	Lee 2020	[19]
	Lee 2022	[20]
	Moharir 2017	[62]
	Nafisah 2019	[69]
	Onu 2019	[37]
	Pokorny 2017	[28]
	Porter 2019	[41]
	Sharan 2021	[45]
	Sharma 2022	[31]
	Szklanny 2019	[70]
	Wang 2019	[54]
	Wang 2019	[55]
	Wijesinghe 2019	[27]
	Wu 2019	[21]
	Zhang 2022	[64]
Neuro Fuzzy Algorithm	Nafisah 2019	[69]
Radial Basis Function Network	Aggarwal 2020	[13]
	Deng 2017	[18]
Random Forest	Aggarwal 2018	[14]
	Aggarwal 2020	[15]
	Chen 2023	[16]
	Chi 2022	[26]
	Manigault 2022	[67]
	McGinnis 2019	[63]
	Suthar 2022	[50]
	Tartarisco 2021	[56]
ResNet	Kotarba 2020	[59]
Statistically Trained Language Model	Alharbi 2018	[51]
Support Vector Machine	Aggarwal 2020	[13]
	Aggarwal 2018	[14]
	Aggarwal 2020	[15]
	Asgari 2021	[22]
	Badreldine 2018	[34]
	Barua 2023	[12]
	Bokov 2015	[47]
	Deng 2017	[18]
	Dubey 2018	[53]
	Gouda 2019	[11]
	Ji 2019	[36]
	Khalilzad 2022	[65]
	Khalilzad 2022	[66]
	Lee 2020	[19]
	MacFarlane 2022	[23]
	Mazic 2015	[48]
	McGinnis 2019	[63]
	Miodonska 2016	[60]
	Nakai 2017	[24]
	Pokorny 2022	[25]
	Pokorny 2017	[28]
	Ribeiro 2020	[33]
	Salehian Matikolaie 2020	[68]
	Sharan 2017	[42]
	Sharan 2019	[44]
	Sharma 2020	[30]
	Suthar 2022	[50]
	Tartarisco 2021	[56]
	Tulics 2018	[57]
	Wang 2019	[55]
	Wu 2019	[21]
Wav2Vec 2.0	Chi 2022	[26]

## Appendix G. Model Accuracy, Sensitivity, and Specificity

**Table A7:** Model Accuracy, Sensitivity, and Specificity.

Model Accuracy, Sensitivity, and Specificity								
Conditon Group	Study	Condition Type	Voice Type	Feature Extraction Methods	Artificial Intelligence Model	Accuracy	Sensitivity	Specificity
Develop-mental	Jayasree 2021	Autism Spectrum Disorder	Voice	Mel Frequency Cepstral Coefficients	Neural Network	100	100	100
	Jayasree 2021	Autism Spectrum Disorder	Voice	RASTA-PLP	Neural Network	100	100	100
	Sharma 2020	Intellectual Disability	Voice	Local Binary Patterns	Support Vector Machine	98.7	99.2	99
	Aggarwal 2018	Intellectual Disability	Voice	Mel Frequency Cepstral Coefficients Linear Predictive Coding	Support Vector Machine	98	97.5	100
	Aggarwal 2020b	Intellectual Disability	Voice	Mel Frequency Cepstral Coefficients Linear Predictive Cepstral Coefficients Spectral Features	Support Vector Machine	98	97.5	100
	Ribeiro 2020	Dyslexia	Voice	Mel Frequency Cepstral Coefficients Spectral Components	Support Vector Machine	94.4	80	100
	Sharma 2022	Intellectual Disability	Voice	Speech Segments	Neural Network	91.9	92.3	91.7
	MacFarlane 2022	Autism Spectrum Disorder	Voice	Cepstral Coefficients Delta Coefficients Harmonic-to-Noise Ratio Pitch and Fundamental Frequency	Automated Language Measures	79.8	82.9	77.3
	Chen 2023	Intellectual Disability	Voice	Mel Frequency Cepstral Coefficients Linear Predictive Cepstral Coefficients	Naïve Bayes	79.6	69	83.2
	Chi 2022	Autism Spectrum Disorder	Voice	Mel Frequency Cepstral Coefficients Spectral Components Zero Crossing Rate	Neural Network	79.3	80.4	79.3
	Cho 2019	Autism Spectrum Disorder	Voice	Mel Frequency Cepstral Coefficients Formant Frequency Pitch and Fundamental Frequency Zero Crossing Rate	Linear Discriminant Analysis	76	76	76
	Asgari 2021	Autism Spectrum Disorder	Voice	Cepstral Coefficients Harmonic-to-Noise-Ratio Spectral Features	Support Vector Machine	74.5	70	79.2
	MacFarlane 2022	Autism Spectrum Disorder	Voice	Cepstral Coefficients Delta Coefficients Harmonic-to-Noise Ratio Pitch and Fundamental Frequency	Support Vector Machine	72.2	68.6	75
	Lee 2020	Autism Spectrum Disorder	Voice	eGeMAPS	Neural Network	70.8	67.6	69.7
	Lee 2022	Autism Spectrum Disorder	Voice	Mel Frequency Cepstral Coefficients	Neural Network	68.2	45.3	68.7
	Nakai 2017	Autism Spectrum Disorder	Voice	Delta Coefficients Pitch and Fundamental Frequency	Support Vector Machine	66	69	61
	Lee 2022	Autism Spectrum Disorder	Voice	Mel Frequency Cepstral Coefficients	Neural Network	61.8	13.1	77.5
Respiratory	Hariharan 2018	Asphyxia	Cry	Linear Predictive Coefficients Non-Linear Entropies Wavelet Packet Transform	Improved Binary Dragonfly Optimization	100	100	100
	Gouda 2019	Wheezing	Respiratory	Mel Frequency Cepstral Coefficients Discrete Wavelet Transform Short Time Fourier Transform	Neural Network	100	100	100
	Mazic 2015	Wheezing	Respiratory	Mel Frequency Cepstral Coefficients	Support Vector Machine	99.9	99.9	99.7
	Amrulloh 2015a	Cough Segments	Cough	Mel Frequency Cepstral Coefficients Shannon Entropy Non-Gaussianity Score Zero Crossing Rate	Linear Discriminant Analysis	97.4	92.8	97.5
	Amrulloh 2018	Wet/Dry Cough	Cough	Mel Frequency Cepstral Coefficients Non-Gaussianity Score Formant Frequency Linear Predictive Cepstral Coefficients Statistical Measures	Neural Network	96.4	96.6	96.5
	Khalilzad 2022a	Respiratory Distress Syndrome	Cry	Cepstral Coefficients Harmonic-to-Noise Ratio	Support Vector Machine	95.3	95	95
	Amrulloh 2015b	Asthma	Cough	Mel Frequency Cepstral Coefficients Shannon Entropy Non-Gaussianity Score Formant Frequency	Neural Network	94.4	88.9	100
	Sharan 2017	Croup	Cough	Mel Frequency Cepstral Coefficients Cochleagram Image Feature	Support Vector Machine	91.2	91.6	88.4
	Sharan 2021	Whooping Cough	Respiratory	Mel Frequency Cepstral Coefficients Cochleagram Image Feature	Neural Network	90.5	85.7	95.2
	Sharan 2019	Croup	Cough	Mel Frequency Cepstral Coefficients Cochleagram Image Feature	Support Vector Machine	86.1	85.3	92.3
	Bokov 2015	Wheezing	Respiratory	Power Spectral Density Peak	Support Vector Machine	84	89	71
	Hee 2019	Asthma	Cough	Mel Frequency Cepstral Coefficients Cepstral Coefficients	Gaussian Mixture Model	83.8	84.8	82.8
	Salehian Matikolaie 2020	Respiratory Distress Syndrome	Cry	Mel Frequency Cepstral Coefficients Cepstral Coefficients Energy	Support Vector Machine	73.8	81.1	60.2
Speech Language	Hariharan 2018	Deafness	Cry	Linear Predictive Coefficients Non-Linear Entropies Wavelet Packet Transform	Improved Binary Dragonfly Optimization	100	100	100
	Barua 2023	Speech Language Impairment	Voice	Wavelet Packet Decomposition	Support Vector Machine	99.9	99.9	99.9
	Kotarba 2020	Speech Language Impairment	Voice	Mel Frequency Cepstral Coefficients Statistical Measures	ResNet	99.5	99	98.6
	Miodonska 2016	Sigmatism	Voice	Mel Frequency Cepstral Coefficients Delta Coefficients	Support Vector Machine	94.5	93	96
	Alharbi 2018	Stuttering	Voice	Mel Frequency Cepstral Coefficients	Statistically-Trained Language Model	94.3	63	32
	Suthar 2022	Speech Disorder	Voice	Landmark Analysis	Linear Discriminant Analysis	93	92	94
	Dubey 2018	Hypernasality	Voice	Mel Frequency Cepstral Coefficients	Support Vector Machine	88	88	88
	Woloshuk 2018	Sigmatism	Voice	Mel Frequency Cepstral Coefficients Spectral Components Statistical Measures	Linear Discriminant Analysis	87.3	91.3	88.6
	Sadeghian 2015	Pediatric Speech Delay	Voice	Mel Frequency Cepstral Coefficients Discrete Cosine Coefficients	Hidden Markov Models	86.1	93.2	52

## Appendix H. Bridge2AI-Voice Consortium List of Authors

**Table A8:** Bridge2AI-Voice Consortium (2022–2023).

Co-Principal Investigators and Module Leads (Level 1)								
Last Name	First Name + Initial	Degree	Institution	Location	Role	Title	ORCID ID	Email
Bensoussan	Yael E.	MD, MSc	University of South Florida	Tampa, FL, USA	Co-PI	Assistant Professor, Department of Otolaryngology—Head & Neck Surgery	0000-0002-1635-8627	yaelbensoussan@usf.edu
Elemento	Olivier	PhD	Weill Cornell Medicine	New-York, NY, USA	Co-PI	Professor of Physiology and Biophysics, Department of Physiology and Biophysics	0000-0002-8061-9617	ole2001@med.cornell.edu
Rameau	Anaïs	MD MSc MS MPhil	Weill Cornell Medicine	New-York, NY, USA	Module Co-Lead, Data Acquisition	Assistant Professor, Department of Otolaryngology—Head & Neck Surgery	0000-0003-1543-2634	anr2783@med.cornell.edu
Sigaras	Alexandros	MSc	Weill Cornell Medicine	New-York, NY, USA	Module Lead, Tools	Assistant Professor of Research in Physiology and Biophysics, Department of Physiology and Biophysics	0000-0002-7607-559X	als2076@med.cornell.edu
Ghosh	Satrajit	PhD	Massachusetts Institute of Technology	Boston, MA, USA	Module Co-lead, Data Acquisition	Principal Research Scientist, Director of the Open Data in Neuroscience Initiative, McGovern Institute, MIT	0000-0002-5312-6729	satra@mit.edu
Powell	Maria E.	PhD, CCC-SLP	Vanderbilt University Medical Center	Nashville, TN, USA	Module Lead, PEDP	Research Assistant Professor, Department of Otolaryngology—Head & Neck Surgery	0000-0002-6643-8991	maria.e.powell@vumc.org
Johnson	Alistair	PhD	University of Toronto	Toronto, Ontario, Canada	Module Lead, Standard	Independent scientist, Assistant Professor		alistair@glowyr.ca
Ravitsky	Vardit	PhD	University of Montreal	Montreal, Quebec, Canada	Module Co-lead, Ethics	Professor, University of Montreal	0000-0002-7080-8801	vardit.ravitsky@umontreal.ca
Bélisle-Pipon	Jean-Christophe	PhD	Simon Fraser University	Burnaby, BC, Canada	Module Co-lead, Ethics	Assistant Professor in Health Ethics	0000-0002-8965-8153	jean-christophe_belisle-pipon@sfu.ca
Dorr	David	MD, MS, FACMI	Oregon Health & Science University	Portland, Oregon, USA	Module Co-lead, SWD	Professor and Vice Chair, Medical Informatics and Clinical Epidemiology	0000-0003-2318-7261	dorrd@ohsu.edu
Payne	Phillip	PhD	Washington University in St Louis	St Louis, MO, USA	Module Co-lead, SWD	Associate Dean and Chief Data Scientist, School of Medicine; Becker Professor and Director, Institute for Informatics, Data Science, and Biostatistics	0000-0002-9532-2998	prpayne@wustl.edu
Hersh	Bill	MD	Oregon Health & Science University	Portland, OR, USA	Skill and Workforce Development	Professor, Medical Informatics and Clinical Epidemiology	0000-0002-4114-5148	hersh@ohsu.edu
